# PHGDH restricts porcine reproductive and respiratory syndrome virus replication by sustaining cellular antioxidant capacity and redox homeostasis

**DOI:** 10.1128/spectrum.00588-26

**Published:** 2026-06-25

**Authors:** Zhangping Yu, Qiongqiong Zhou, Peng Gao, Yongning Zhang, Xinna Ge, Jun Han, Xin Guo, Lei Zhou, Hanchun Yang

**Affiliations:** 1 State Key Laboratory of Veterinary Public Health and Safety, College of Veterinary Medicine, China Agricultural University 34752, Beijing, People’s Republic of China; 2 Key Laboratory of Animal Epidemiology of Ministry of Agriculture and Rural Affairs, College of Veterinary Medicine, China Agricultural University 34752, Beijing, People’s Republic of China; Shandong First Medical University, Jinan, Shandong, China

**Keywords:** PRRSV, PHGDH, oxidative stress, ROS, mitochondrial damage, viral replication

## Abstract

Porcine reproductive and respiratory syndrome virus (PRRSV) remains a major threat to the swine industry. Although redox imbalance and oxidative stress are known to favor PRRSV infection, the host determinants that preserve redox homeostasis during PRRSV replication remain insufficiently defined. Here, we identify phosphoglycerate dehydrogenase (PHGDH), the rate-limiting enzyme of the *de novo* serine synthesis pathway, as a novel restriction factor for PRRSV. We found that PRRSV infection downregulated PHGDH at both mRNA and protein levels. PHGDH knockdown significantly enhanced PRRSV replication in MARC-145 cells and primary porcine alveolar macrophages, whereas ectopic PHGDH expression suppressed viral replication. Specifically, PHGDH depletion promoted PRRSV replication and release, but not attachment or internalization. Catalytically inactive PHGDH mutants retained PRRSV-restriction activity comparable to wild type, indicating a non-canonical, catalysis-independent function. Mechanistically, PHGDH depletion coordinately reduced antioxidant defense genes, oxidized cellular reducing pools, and increased intracellular ROS and mitochondrial superoxide. Importantly, antioxidant treatment with N-acetylcysteine, reduced glutathione, or Mito-TEMPO abolished the proviral effect of PHGDH knockdown, establishing oxidative stress as an essential mediator of enhanced PRRSV replication. PHGDH depletion also caused mitochondrial structural and functional abnormalities and promoted glycolytic features; however, antioxidants failed to restore mitochondrial integrity, and glycolysis inhibition did not reverse the elevated viral replication in PHGDH-depleted cells, indicating that oxidative stress—rather than glycolysis or secondary mitochondrial injury—drives the proviral phenotype. Collectively, our data uncover a PHGDH–redox axis that limits PRRSV replication and highlight redox homeostasis as a tractable host process for antiviral intervention.

**IMPORTANCE:**

Porcine reproductive and respiratory syndrome virus is a leading cause of respiratory disease and reproductive failure in pigs, creating major economic losses worldwide. Effective control remains challenging, so identifying host processes that naturally limit infection can reveal new intervention opportunities. Our study shows that a host metabolic enzyme, phosphoglycerate dehydrogenase, helps cells maintain antioxidant capacity and redox balance, and this cellular “buffer” restricts porcine reproductive and respiratory syndrome virus replication. When this protection is weakened, oxidative stress rises and the virus benefits; restoring redox balance counters this effect. These findings highlight redox homeostasis as an important, druggable host pathway and suggest that strengthening the cell’s own stress defenses may complement existing strategies to reduce virus burden and improve swine health.

## INTRODUCTION

Porcine reproductive and respiratory syndrome (PRRS) is one of the most economically important infectious diseases of swine worldwide, characterized by reproductive failure in sows and respiratory disease in all age pigs (1–3). PRRSV, the etiologic agent, is an enveloped, positive-sense RNA virus in the family *Arteriviridae* (order *Nidovirales*) and exhibits extensive genetic diversity and immune evasion, which complicate vaccine-mediated control and contribute to persistent circulation in pig populations (4, 5). With the rapid progress in studies of virus-host interplay, our understanding of the molecular events governing viral infection has deepened substantially (6–8). Therefore, elucidating the mechanisms of virus-host interactions may contribute to developing novel strategies for preventing and controlling epidemic strains.

A growing body of work supports a functional link between viral infection and cellular redox homeostasis (9). Reactive oxygen species (ROS) are generated as by-products of metabolism and also function as signaling molecules; excessive ROS causes oxidative stress that can reshape innate immunity, cell survival pathways, and viral replication fitness (10–15). PRRSV infection is associated with oxidative stress and can exploit redox signaling to optimize replication. For example, PRRSV GP5 promotes mitochondrial ROS production that activates autophagy and supports viral replication (16). In parallel, host antioxidant pathways such as the Nrf2/HO-1 axis can exert antiviral effects against PRRSV, and PRRSV-encoded factors may counteract such antioxidant defenses (17, 18). These findings underscore that redox balance is not merely a bystander outcome of infection but can be a determinant of PRRSV replication and pathogenesis.

Viruses commonly reprogram host metabolism to satisfy biosynthetic and bioenergetic demands (19). Beyond supplying macromolecular precursors, metabolic networks also govern redox buffering capacity through reduced pyridine nucleotides (NADH and NADPH) and thiol-based antioxidants such as glutathione (10–13). The *de novo* serine synthesis pathway branches from glycolysis and provides serine and glycine that fuel one-carbon metabolism, nucleotide synthesis, and antioxidant programs, in part by supporting glutathione biosynthesis and NADPH generation (20, 21). PHGDH catalyzes the first and rate-limiting step of this pathway and has been extensively studied in cancer metabolism and redox control (22). Notably, PHGDH can influence cellular resilience to oxidative stress by sustaining reducing equivalents and antioxidant capacity (23, 24). However, whether and how PHGDH contributes to host-PRRSV interactions has not been defined.

In this study, we demonstrate that PHGDH suppresses PRRSV replication by maintaining cellular antioxidant capacity and redox homeostasis. Using loss- and gain-of-function approaches in MARC-145 cells, coupled with transcriptomics, redox metabolite measurements, and functional antioxidant rescue experiments, we show that PHGDH depletion induces oxidative stress that is necessary for enhanced PRRSV replication. We further observe mitochondrial abnormalities induced by PHGDH depletion that are not rescued by antioxidants, suggesting separable roles of PHGDH in redox control and mitochondrial homeostasis. Together, our findings highlight a PHGDH–redox axis that restricts PRRSV and provide mechanistic insight into how metabolic redox homeostasis intersects with *Arterivirus* replication.

## RESULTS

### PHGDH is a host-restricted factor that antagonizes PRRSV proliferation

**Fig 1:**
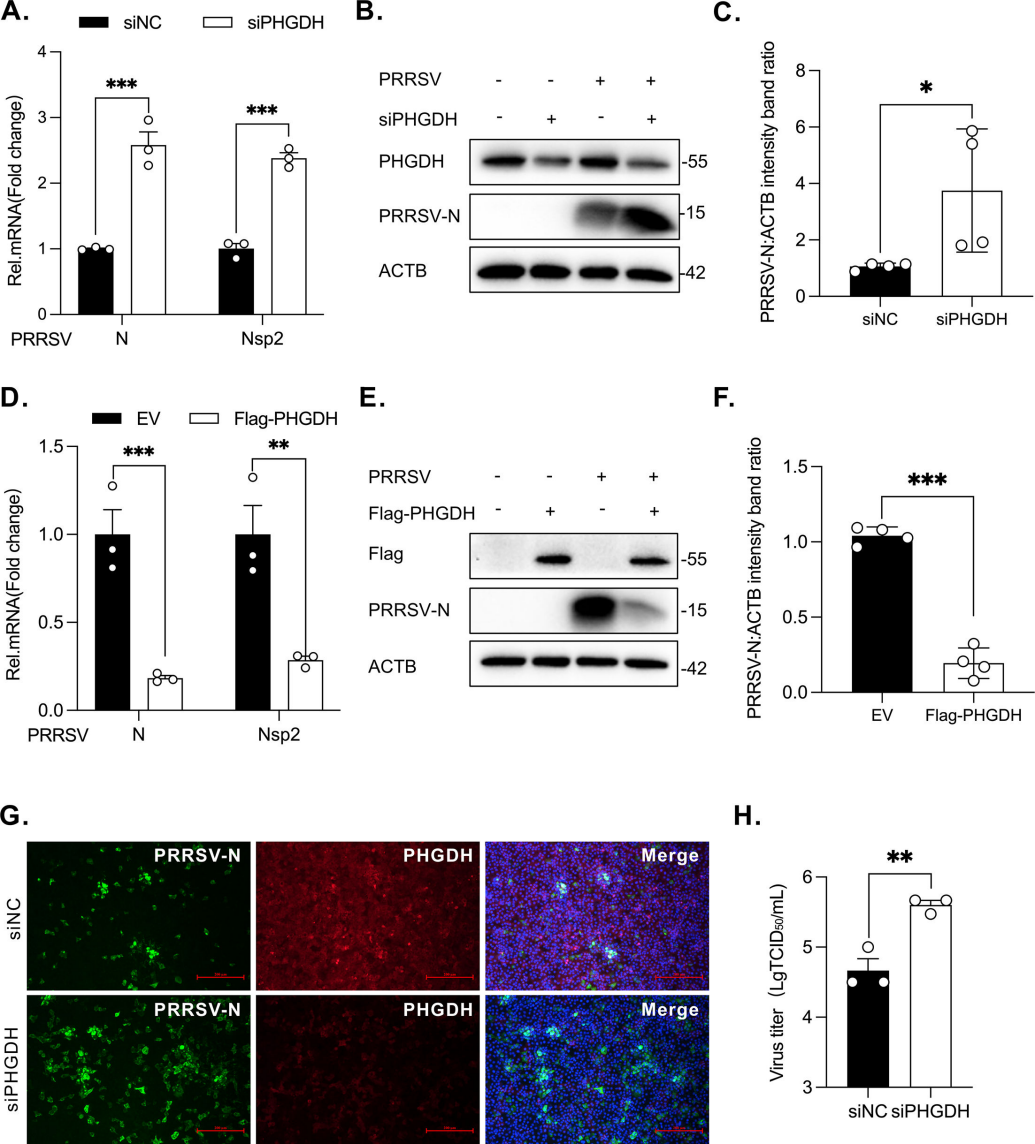
PHGDH restricts PRRSV replication in MARC-145 cells. (**A–C**) MARC-145 cells were transfected with control siRNA (siNC) or PHGDH-targeting siRNA (siPHGDH) and infected with PRRSV (MOI = 0.1). Viral N and nsp2 mRNA were quantified by reverse transcription-quantitative polymerase chain reaction (RT-qPCR) (**A**), N protein by Western blot (**B**), and PRRSV N protein band intensities normalized to loading control via ImageJ (**C**). (**D–F**) Cells were transfected with empty vector (EV) or Flag-PHGDH and infected with PRRSV (MOI = 0.5). Viral N and nsp2 mRNA were quantified by RT-qPCR (**D**), N protein by Western blot (**E**), and PRRSV N protein band intensities normalized to loading control via ImageJ (**F**). (**G**) Immunofluorescence staining of PRRSV N (green) and PHGDH (red) in PRRSV-infected siNC versus siPHGDH cells; nuclei were counterstained with DAPI (blue). Scale bars, 200 μm. (**H**) Virus titers in siNC and siPHGDH cells were determined by TCID_50_ and expressed as log10TCID_50_/mL. Data are mean ± SD of three independent experiments; each dot represents one independent replicate (*n* ≥ 3). Statistical significance was assessed by a two-tailed Student’s *t*-test, *P < 0.05; **P < 0.01; ***P < 0.001.

To determine whether PHGDH influences PRRSV replication, we first depleted PHGDH using two independent siRNAs (siPHGDH-1 and siPHGDH-2) and confirmed knockdown by immunoblotting (Fig. S1A). Then, MARC-145 cells were transfected with siPHGDH-1 and infected with PRRSV (MOI = 0.1) for 24 h. Viral mRNA (N and nsp2 genes), N protein, viral titers, and immunofluorescence signal of PRRSV all indicated that PHGDH knockdown significantly increased PRRSV proliferation, with titers almost 10-fold higher (Fig. 1A, B, C, G, and H). Accordingly, the overexpression of PHGDH suppressed the viral replication in MARC-145 cells (Fig. 1D through F). Importantly, the proviral effect of PHGDH depletion was further confirmed in primary porcine alveolar macrophages (PAMs), the natural target cells of PRRSV (Fig. S1B through D).

Next, to examine whether PRRSV infection alters PHGDH expression, MARC-145 cells were infected with PRRSV, and PHGDH expression was determined by reverse transcription-quantitative polymerase chain reaction (RT-qPCR) and Western blot. The results showed that PRRSV infection suppressed both PHGDH gene transcription and protein expression (Fig. S1E through G). Together, these data identify PHGDH as a host restriction factor that is downregulated by PRRSV during infection and suggest that PHGDH depletion creates a cellular state permissive for viral propagation.

### PHGDH affects PRRSV intracellular replication and virion release, but not viral attachment or internalization

**Fig 2:**
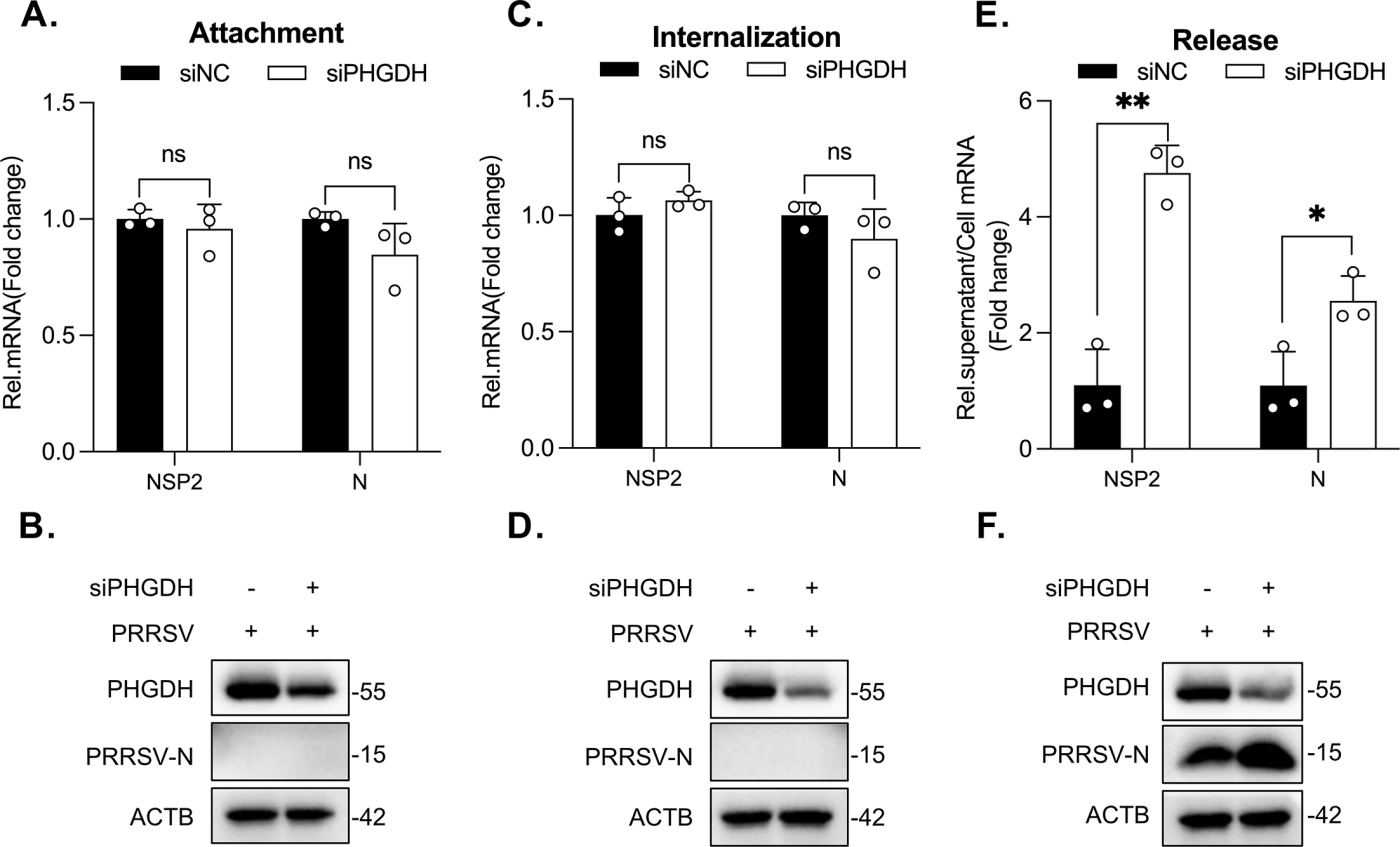
PHGDH affects PRRSV replication and release but has no effect on viral attachment or internalization. (**A–F**) MARC-145 cells were transfected with control siRNA (siNC) or PHGDH-targeting siRNA (siPHGDH) for 24 h and subjected to stage-specific PRRSV infection assays. (**A**) Attachment: cells were incubated with PRRSV (MOI = 1) at 4°C for 1 h, washed to remove unbound virions, and cell-bound viral nsp2 and N RNA were quantified by RT-qPCR. (**B**) Western blot of PHGDH and PRRSV-N protein in the attachment assay; ACTB was used as the loading control. (**C**) Internalization: after 1 h binding at 4°C, cells were shifted to 37°C for 1 h; surface-bound virions were removed by trypsinization, and internalized viral nsp2 and N RNA were quantified by RT-qPCR. (**D**) Western blot of PHGDH and PRRSV-N protein in the internalization assay. (**E**) Release: cells were infected with PRRSV (MOI = 1) for 24 h, and supernatant and cell pellets were collected separately; viral release efficiency was quantified by calculating the ratio of the 2^−ΔΔCt^ value of viral RNA detected in the supernatant to the 2^−ΔΔCt^ value of intracellular viral RNA. (**F**) Western blot of intracellular PHGDH and PRRSV-N protein at the release time point. Data are mean ± SD from three independent biological replicates (*n* ≥ 3). Statistical significance was assessed by a two-tailed Student’s *t*-test. ns, not significant; *P < 0.05; **P < 0.01.

To define which stage of the PRRSV life cycle is affected by PHGDH, we performed stage-specific assays in siNC- and siPHGDH-transfected MARC-145 cells, examining attachment (4°C binding), internalization (37°C, 1 h post-binding with surface-virion removal), and release (extracellular viral RNA at 24 h post-infection). PHGDH knockdown caused no significant change in viral attachment (Fig. 2A) or internalization (Fig. 2C). In contrast, PHGDH knockdown markedly increased the supernatant-to-cell ratio of viral RNA, indicating enhanced release (Fig. 2E). The efficiency of PHGDH knockdown was verified by Western blot at each assay (Fig. 2B, D, and F). Together with the enhanced intracellular viral mRNA and N protein observed at 24 h post-infection in PHGDH-knockdown cells (Fig. 1A and C), these results indicate that PHGDH restricts PRRSV at the post-entry stages of intracellular replication and virion release, but not at attachment or internalization.

### PHGDH depletion suppresses antioxidant programs and oxidizes cellular reducing pools

**Fig 3:**
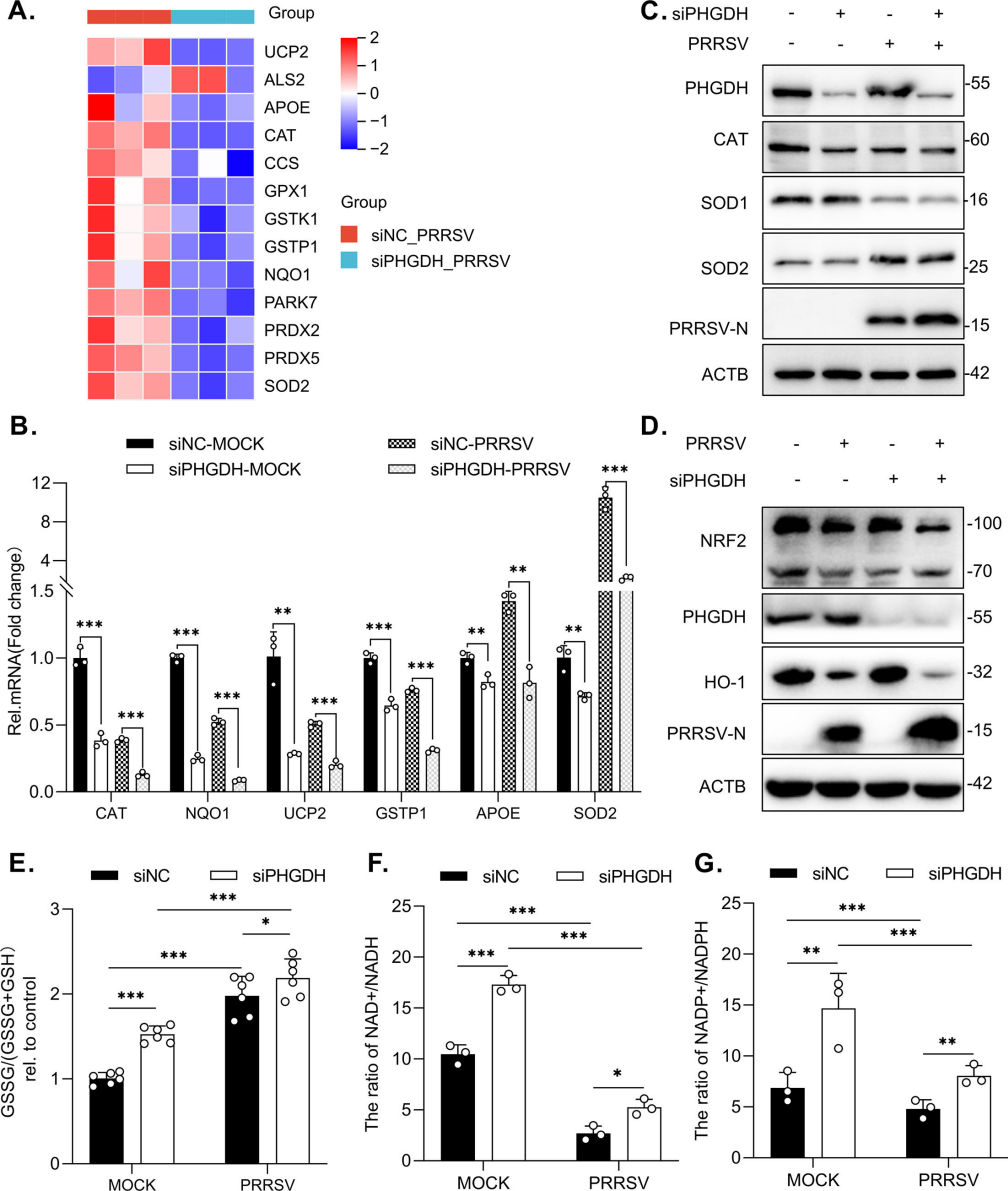
PHGDH depletion downregulates antioxidant programs and reduces reducing equivalents during PRRSV infection. (**A**) Heatmap of representative antioxidant/redox-related genes from RNA-seq comparing PRRSV-infected siNC and siPHGDH groups. (**B**) RT-qPCR validation of selected antioxidant genes (e.g., CAT, NQO1, UCP2, GSTP1, APOE, and SOD2) in mock- or PRRSV-infected cells transfected with siNC or siPHGDH. (**C**) Immunoblot analysis of the expression of PHGDH and antioxidant proteins (CAT, SOD1, and SOD2) in siNC- or siPHGDH-transfected cells with or without PRRSV infection. (**D**) Immunoblot analysis of the expression of Nrf2 and HO-1 in the indicated groups. (**E**) Cellular glutathione redox state expressed as GSSG/(GSSG + GSH). (**F**) NAD+/NADH ratio. (**G**) NADP+/NADPH ratio. Data are mean ± SD of three independent experiments; each dot represents one independent replicate (*n* ≥ 3). Statistical significance was determined using multiple *t*-tests (for panel B) and the two-tailed Student’s *t*-test (for panels E–G), *P < 0.05; **P < 0.01; and ***P < 0.001.

To explore the mechanism of PHGDH downregulation on PRRSV proliferation, the transcriptome analysis for the PRRSV-infected and/or PHGDH-knockdown cells was carried out. RNA-seq analysis of PRRSV-infected PHGDH-knockdown cells revealed broad downregulation of antioxidant and redox-associated genes, including CAT, NQO1, UCP2, GSTP1, APOE, and SOD2 (Fig. 3A). RT-qPCR validated downregulation of representative antioxidant genes (CAT, NQO1, UCP2, GSTP1, APOE, and SOD2) (Fig. 3B), and immunoblotting confirmed reduced antioxidant proteins (CAT, SOD1, and SOD2) (Fig. 3C; S2A through C). The Nrf2/HO-1 pathway is an intracellular antioxidant defense system, which is inhibited during PRRSV infection, thereby inducing oxidative stress to facilitate viral replication (17). Notably, although PHGDH depletion significantly reduced the expression of Nrf2 and HO-1 during PRRSV infection, it did not alter their basal levels in normal, uninfected cells (Fig. 3D; Fig. S2D and E). Immunofluorescence further demonstrated that PHGDH knockdown does not alter the nuclear/cytoplasmic distribution of Nrf2 in mock cells (Fig. S2F). These results suggest that PHGDH does not affect basal Nrf2 localization but may impair the Nrf2/HO-1 antioxidant response during PRRSV infection.

We next quantified redox metabolite indices. The GSSG/(GSSG + GSH) ratio, the NAD+/NADH ratio, and the NADP+/NADPH ratio were markedly elevated in the PHGDH-knockdown cells (Fig. 3E through G), indicating depletion of reduced pyridine nucleotide pools. To determine whether the antiviral activity of PHGDH requires its serine-synthesis catalytic activity, we generated two enzymatically inactive PHGDH mutants on the Flag-PHGDH-WT backbone by site-directed mutagenesis—Flag-PHGDH-R236E and Flag-PHGDH-V425M—and compared their effects with wild-type Flag-PHGDH (Flag-PHGDH-WT) on PRRSV replication. The result showed that the restriction of PRRSV by PHGDH does not require its canonical serine-synthesis catalytic activity (Fig. S2G and H). In summary, these data demonstrate that PHGDH depletion induces a global redox imbalance characterized by weakened antioxidant gene expression and oxidized intracellular reducing equivalents.

### PHGDH knockdown elevates intracellular ROS and mitochondrial ROS

**Fig 4:**
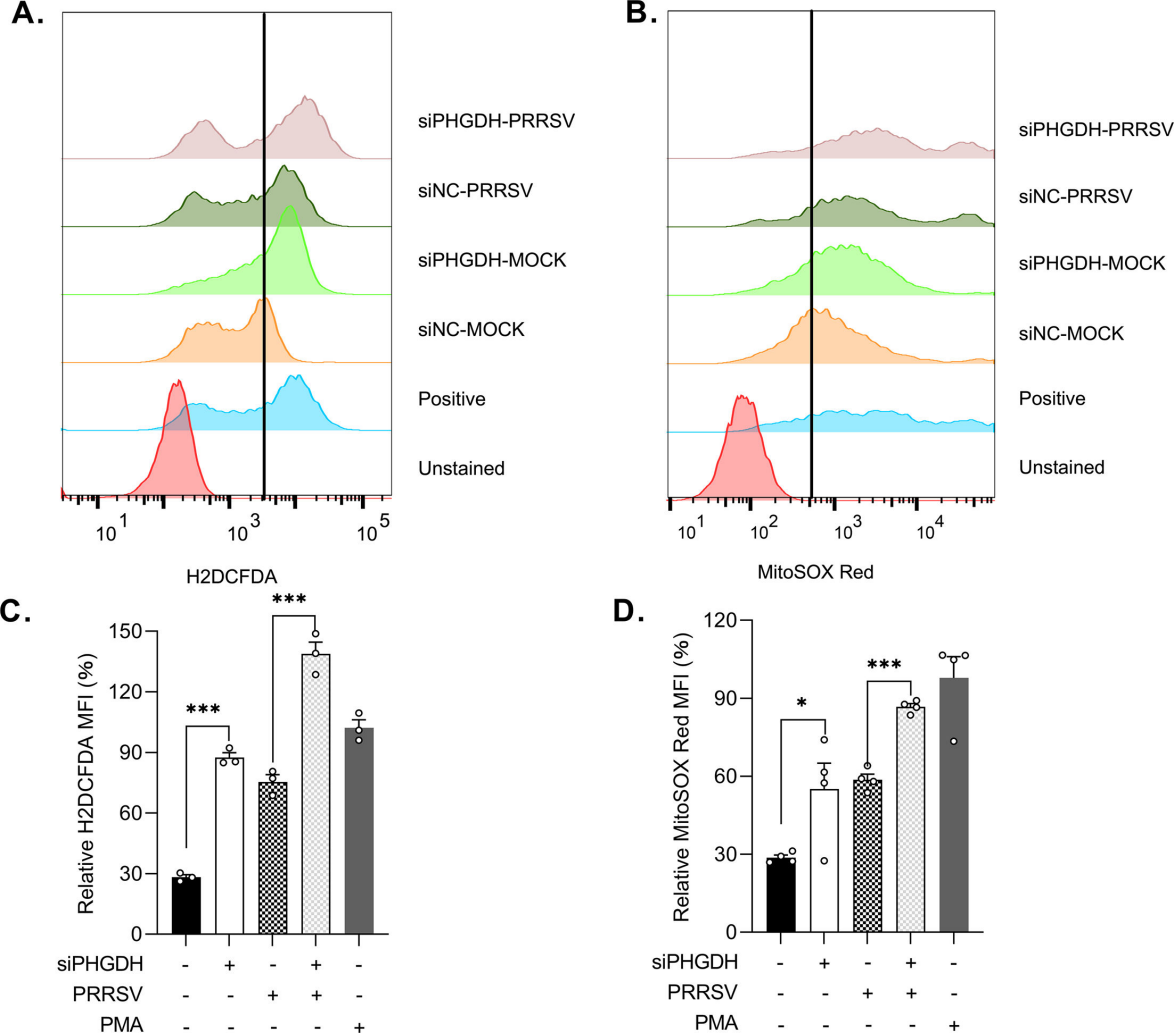
PHGDH knockdown elevates intracellular ROS and mitochondrial ROS. (**A**) Representative flow cytometry histograms of intracellular ROS measured by H2DCFDA staining in mock- or PRRSV-infected cells transfected with siNC or siPHGDH. Unstained and positive-control samples are shown for gating. PMA, positive control. (**B**) Representative flow cytometry histograms of mROS measured by MitoSOX Red staining in the indicated groups. (**C**) Quantification of H2DCFDA mean fluorescence intensity (MFI). (**D**) Quantification of MitoSOX Red MFI. Data are mean ± SD of three independent experiments; each dot represents one independent replicate (*n* ≥ 3). Statistical significance was assessed by a two-tailed Student’s *t*-test, *P < 0.05; ***P < 0.001.

Given the observed reduction in antioxidant capacity and reducing equivalents, intracellular reactive oxygen species and mitochondrial reactive oxygen species (mROS) levels were measured via flow cytometry. H2DCFDA was used as an indicator of intracellular ROS, whereas mROS levels can be quantified by mitochondrial superoxide (MitoSOX) assay (25). In both mock- and PRRSV-infected cells, PHGDH knockdown significantly increased intracellular ROS and mROS (Fig. 4A through D). We also assessed H2DCFDA fluorescence intensity by microscopy and found that siPHGDH cells exhibited markedly higher fluorescence than siNC cells, supporting enhanced ROS reactivity upon PHGDH depletion (Fig. S3A and B). In addition, both H2DCFDA and MitoSOX fluorescence distributions shifted toward higher intensity upon PHGDH depletion (Fig. S3C and D). These results demonstrate a marked increase in both total intracellular ROS and mROS in the PHGDH-knockdown cells, indicating an elevated oxidative stress burden within cells, with mitochondria emerging as a site of ROS accumulation.

### PHGDH knockdown induces antioxidant-resistant mitochondrial injury and aberrant fission signaling

**Fig 5:**
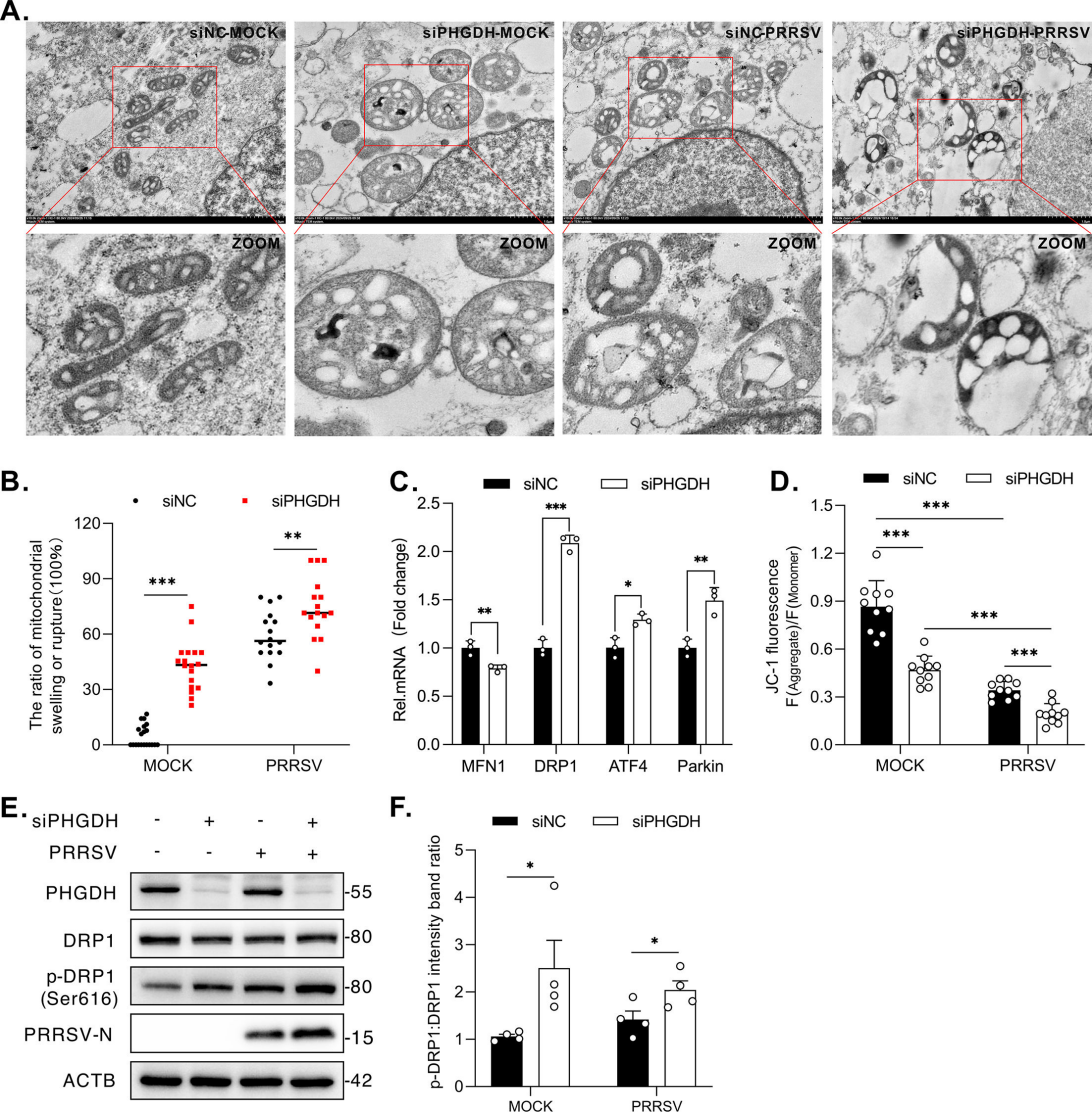
PHGDH depletion triggers mitochondrial structural and functional injury and promotes DRP1-dependent fission signaling. (**A**) Transmission electron microscopy of mitochondria in PHGDH-knockdown MARC-145 cells infected with PRRSV (MOI = 1) or mock infection at 24 h post-infection. Red boxes indicate regions shown at higher magnification (ZOOM). (**B**) Bottom panel shows quantification of damaged mitochondria (≥15 fields per condition from three experiments). Scale bars, 1 μm. Each dot represents a quantified field. (**C**) RT-qPCR analysis of mitochondrial dynamics and stress-associated genes (e.g., MFN1, DRP1, ATF4, and Parkin) in siNC and siPHGDH cells. (**D**) Mitochondrial membrane potential (Δψm) assessed by JC-1 staining and expressed as aggregate/monomer fluorescence ratio. (**E**) Immunoblot analysis of DRP1 and phospho-DRP1 (Ser616) in the indicated groups. (**F**) Densitometric quantification of p-DRP1/DRP1 ratios. Data are mean ± SD of three independent experiments; each dot represents one independent replicate (*n* ≥ 3). Statistical significance was determined using multiple *t*-tests (for panel C) and the two-tailed Student’s *t*-test (for panels **B**, **D**, and **F**), *P < 0.05; **P < 0.01; and ***P < 0.001.

Considering that PHGDH knockdown induced mROS production, it was hypothesized that PHGDH might be relevant to mitochondrial homeostasis. To investigate the effect of PHGDH knockdown on the ultrastructural changes of mitochondria, transmission electron microscopy analysis was performed on mitochondria from PHGDH-knockdown cells and control. As shown in Fig. 5A, cells in mock- or PRRSV-infected PHGDH-knockdown groups displayed pronounced mitochondrial swelling, disrupted cristae, and fragmented mitochondrial structures compared with controls. In control cells, most mitochondria appeared normal, with a rod-like morphology and clearly defined cristae. Further quantification confirmed a higher proportion of damaged mitochondria in the cytoplasm of PHGDH-knockdown cells (Fig. 5B). To identify whether downregulation of PHGDH induced mitochondrial dysfunction, RT-qPCR analysis showed that the transcripts associated with mitochondrial dynamics and quality control were altered: DRP1, ATF4, and Parkin were upregulated, while the fusion factor MFN1 was reduced, suggesting a shift toward mitochondrial fragmentation and stress responses (Fig. 5C). JC-1 staining can be used to monitor changes in mitochondrial membrane potential (Δψm) in response to various stimuli. Under normal conditions, JC-1 forms aggregates and fluoresces red, indicating high Δψm. However, in cells with low Δψm, JC-1 remains monomeric and fluoresces green. Measurement of the red-to-green fluorescence ratio revealed that PHGDH knockdown led to a decrease in the ratio, suggesting the loss of Δψm (Fig. 5D). In addition, DRP1 phosphorylation is required for mitochondrial fission, and immunoblot analysis showed that PHGDH knockdown promoted DRP1 phosphorylation at Ser616 (Fig. 5E and F). The above results indicate that the downregulation of PHGDH induced mitochondrial damage.

Interestingly, the loss of Δψm and the DRP1 phosphorylation were not attenuated when oxidative stress was inhibited with the antioxidant N-acetylcysteine (NAC) (Fig. S4A through C), suggesting that mitochondrial damage is not simply a secondary consequence of ROS accumulation. Consistent with this possibility, PHGDH displayed partial mitochondrial co-localization in confocal microscopy (Fig. S4D and E). Overall, these data demonstrate that PHGDH is essential for maintaining mitochondrial structure and function, and that PHGDH supports mitochondrial integrity through an antioxidant-independent mechanism.

### PHGDH knockdown induces a glycolytic shift, but glycolysis is dispensable for enhanced viral replication

**Fig 6:**
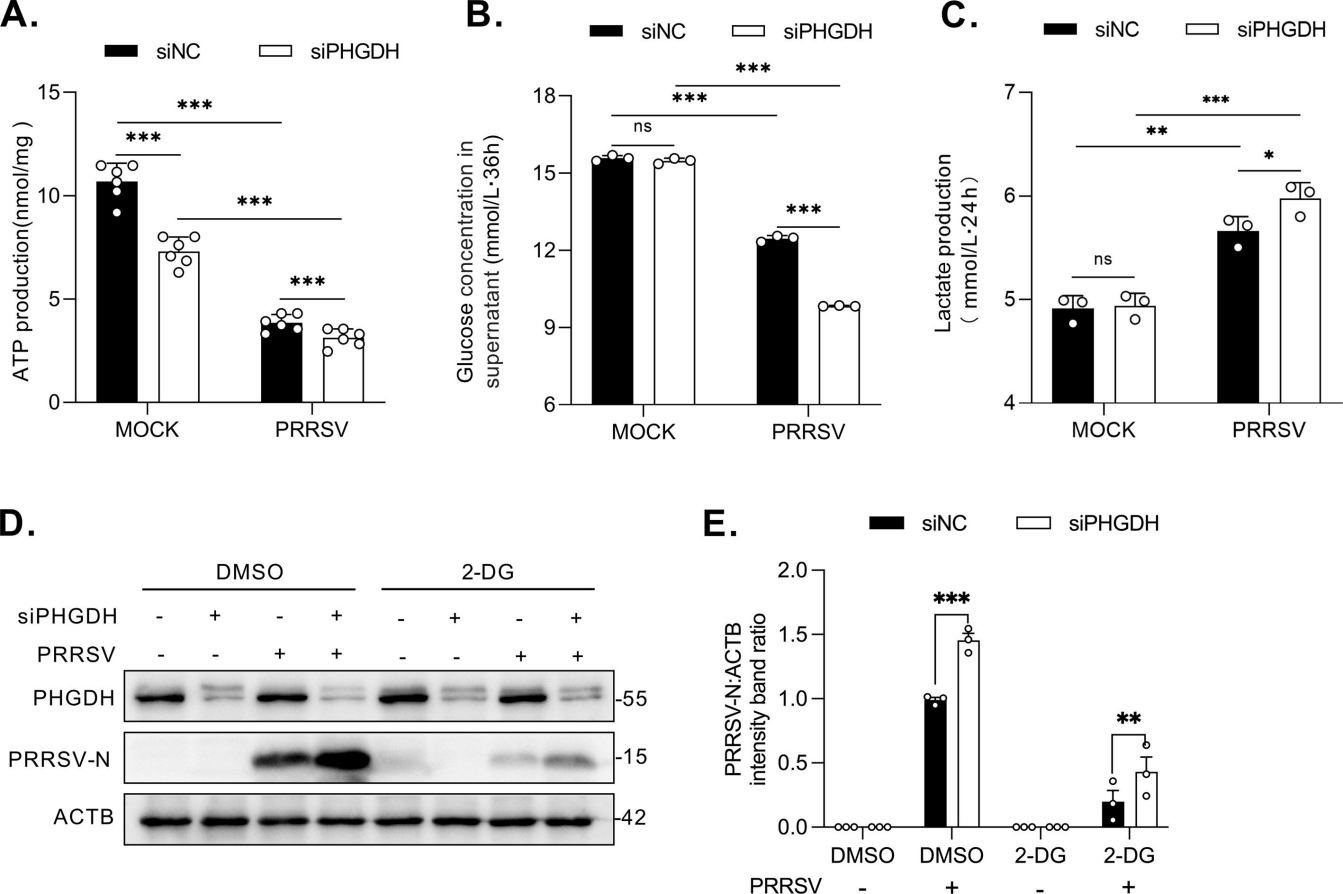
PHGDH knockdown induces an ATP deficit and a glycolytic shift, but glycolysis inhibition does not abolish enhanced PRRSV replication. (**A**) Intracellular ATP levels in mock- or PRRSV-infected siNC versus siPHGDH cells. (**B**) Glucose concentration/consumption in culture supernatants. (**C**) Lactate production in culture supernatants. (**D**) PRRSV N protein expression in siNC- or siPHGDH-transfected cells cultured with or without 10 μM 2-DG. (**E**) Quantification of panel D. Band intensities were quantified by ImageJ. Data are mean ± SD of three independent experiments; each dot represents one independent replicate (*n* ≥ 3). Statistical significance was determined using the two-tailed Student’s *t*-test (for panels **A**–**E**), *P < 0.05; **P < 0.01; and ***P < 0.001.

It was reported that PRRSV infection induced mitochondrial dysfunction and significantly reduced intracellular ATP production (26). Consistent with previous reports, our results demonstrated that PRRSV infection significantly reduced ATP production. Furthermore, ATP levels were reduced in PHGDH-knockdown cells (Fig. 6A). In addition, as demonstrated above, the elevated NAD+/NADH and NADP+/NADPH ratios suggest that the downregulation of PHGDH disrupted oxidative phosphorylation (OXPHOS) in mitochondria. As a primary site of ATP production, cellular energy metabolism in mitochondria is altered by injury. Therefore, we investigated the alteration of OXPHOS and glycolytic processes in the PHGDH-knockdown cells. As shown in Fig. 6B and C, in PRRSV-infected cultures, PHGDH depletion increased glucose consumption (lower residual glucose in supernatants) and elevated lactate secretion, consistent with increased glycolytic flux. We then asked whether enhanced glycolysis mediates the proviral effect of PHGDH knockdown. Treatment with the glycolysis inhibitor 2-deoxy-D-glucose (2-DG) reduced overall viral protein abundance; however, it did not reverse the increased PRRSV N protein level observed in PHGDH-depleted cells (Fig. 6D through E). These findings indicate that glycolysis is not the dominant mechanism by which PHGDH depletion enhances PRRSV replication.

### Oxidative stress is required for PHGDH knockdown–enhanced PRRSV replication

**Fig 7:**
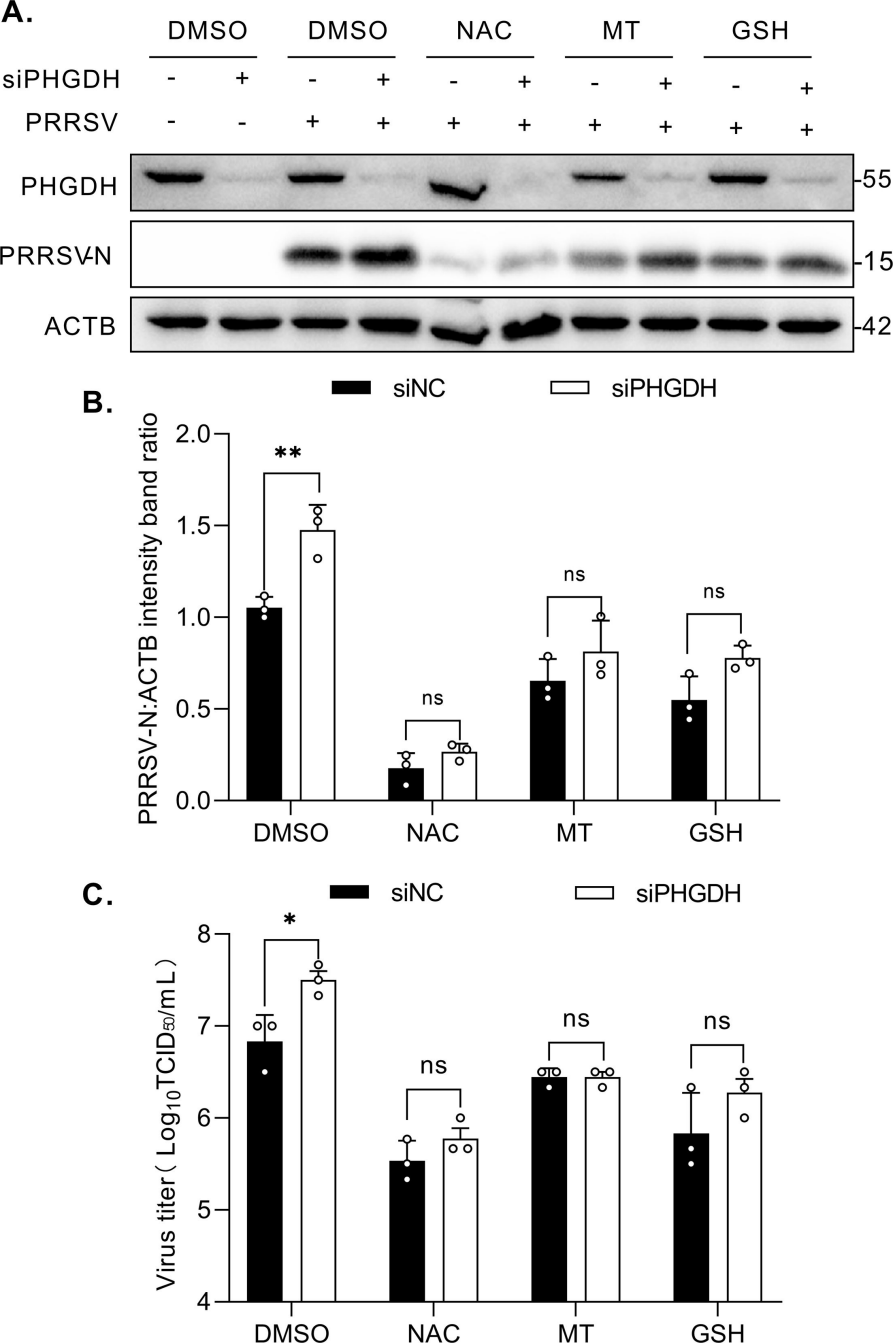
Oxidative stress is required for PHGDH knockdown–enhanced PRRSV replication. (**A–C**) Rescue of PHGDH knockdown-mediated PRRSV enhancement by antioxidants. Cells transfected with siPHGDH or siNC were treated with 10 mM NAC, 10 μM MT, or 5 mM GSH and then infected with PRRSV (MOI = 0.1). Viral N protein was detected by Western blot (**A**) and quantified by ImageJ (**B**). Viral titer was determined by TCID_50_ assay and expressed as log10TCID_50_/mL. (**C**). Data are mean ± SD of three independent experiments; each dot represents one independent replicate (*n* ≥ 3). Statistical significance was determined using multiple *t*-tests. ns, not significant; *P < 0.05; and **P < 0.01.

To directly test whether oxidative stress is required for the proviral phenotype, we treated cells with antioxidants. N-acetylcysteine, reduced glutathione (GSH), and the mitochondrial-targeted ROS scavenger Mito-TEMPO (MT) all significantly reduced PRRSV N protein expression and decreased viral titers. Importantly, these antioxidant treatments largely eliminated the replication advantage conferred by PHGDH knockdown, resulting in comparable levels of PRRSV replication between siNC and siPHGDH groups (Fig. 7A through C). Having established that ROS is necessary for the enhanced PRRSV replication observed upon PHGDH depletion, we next asked whether exogenous induction of oxidative stress is sufficient to promote PRRSV replication in the presence of PHGDH. MARC-145 cells were treated with the classical ROS inducer PMA (5 nM) and infected with PRRSV. Immunoblot analysis demonstrated that PMA treatment significantly increased PRRSV N protein expression (Fig. S5A and B), confirming that oxidative stress alone is sufficient to create a proviral environment. These data establish oxidative stress as a necessary mediator of PHGDH knockdown–enhanced PRRSV replication, separating the proviral redox axis from the NAC-resistant mitochondrial injury axis.

## DISCUSSION

In this study, we identify the serine biosynthesis enzyme PHGDH as a host factor that restricts PRRSV replication and dissect a mechanistic link to cellular redox homeostasis. Our data support five main findings: (i) PRRSV infection naturally downregulates PHGDH expression at both mRNA and protein levels; (ii) PHGDH depletion robustly promotes PRRSV replication, whereas PHGDH overexpression suppresses it, and this antiviral activity is independent of the serine-synthesis catalytic activity of PHGDH; (iii) the proviral effect of PHGDH loss is restricted to PRRSV intracellular replication and virion release rather than attachment or internalization; (iv) PHGDH knockdown compromises antioxidant gene expression and reducing equivalent pools, resulting in elevated ROS/mROS that are necessary for enhanced viral replication; and (v) PHGDH depletion triggers mitochondrial structural injury and fission signaling that cannot be reversed by NAC, suggesting an additional antioxidant-independent role for PHGDH in mitochondrial integrity.

PHGDH sits at the intersection of glycolysis, amino acid metabolism, and redox regulation. By catalyzing the committed step of *de novo* serine synthesis, PHGDH supports the generation of serine-derived metabolites, including glycine and GSH, and supplies one-carbon units to folate cycles that generate NADPH for reductive biosynthesis and antioxidant defense (20, 22, 24, 27, 28). Consistent with these established links, PHGDH depletion in our system led to reduced NADH and NADPH availability (increased NAD+/NADH and NADP+/NADPH ratios) and to a shift toward an oxidized glutathione pool (increased GSSG/[GSSG + GSH]). Importantly, transcriptome analysis revealed broad downregulation of antioxidant genes (CAT, NQO1, GSTP1, and others), providing a convergent explanation for the redox imbalance. Together, these results position PHGDH as an upstream determinant of antioxidant capacity during PRRSV infection.

Why does oxidative stress promote PRRSV replication? Moderate ROS elevation can facilitate replication of diverse RNA viruses by modulating redox-sensitive signaling nodes that control autophagy, inflammatory responses, and innate antiviral pathways (15, 29). In PRRSV infection, mitochondrial ROS has been reported to promote replication by triggering autophagy downstream of enhanced ER–mitochondria contact and Ca^2+^ uptake driven by GP5 (16). Moreover, the Keap1–Nrf2 axis has emerged as an antiviral pathway for PRRSV; activation of Nrf2/HO-1 signaling restricts replication, whereas PRRSV nsp5 can degrade Nrf2 and dampen antioxidant gene induction (17, 18, 30). Within this framework, PHGDH depletion likely lowers the threshold for redox imbalance, facilitating ROS/mROS accumulation upon infection. Consistent with a causal requirement, scavenging ROS with NAC, Mito-TEMPO, or exogenous GSH abolished the replication enhancement conferred by PHGDH knockdown. These findings argue that oxidative stress is not merely a byproduct of the infection/metabolic state but a functional driver of PRRSV replication in the context of PHGDH loss.

Interestingly, although PHGDH depletion increased both cytosolic ROS and mROS, the inability of NAC to rescue mitochondrial membrane potential and DRP1 phosphorylation suggests that mitochondrial injury is not simply a downstream consequence of ROS accumulation. PHGDH has recently been shown to localize to the inner mitochondrial membrane and promote mitochondrial translation and respiratory metabolism through a non-canonical, catalytic-independent function (31). Our co-localization data support mitochondrial association of PHGDH in MARC-145 cells. We therefore speculate that PHGDH depletion may directly impair mitochondrial homeostasis (e.g., OXPHOS complex biogenesis/translation, membrane organization, or nucleotide exchange), thereby promoting depolarization and a shift toward fission signaling. Such primary mitochondrial defects could increase mROS production; however, while antioxidants can neutralize ROS to suppress the proviral phenotype, they would not necessarily repair structural/functional mitochondrial lesions caused by loss of PHGDH. Future work testing PHGDH mutants that separate enzymatic activity from mitochondrial localization/interaction partners will be important to resolve this dual role.

PRRSV is known to remodel cellular metabolism, and glycolysis has been implicated as a proviral pathway in several studies (32–34). In our experiments, PHGDH depletion reduced cellular ATP and promoted glucose consumption and lactate production in infected cells, consistent with reduced OXPHOS and a compensatory glycolytic shift. However, inhibiting glycolysis with 2-DG did not abolish the replication advantage in PHGDH-depleted cells, suggesting that the key driver is oxidative stress rather than glycolytic flux *per se*. One possibility is that glycolysis contributes to baseline PRRSV replication in MARC-145 cells, but the additional replication gain caused by PHGDH loss is mediated by redox imbalance. Dissecting the relative contributions of metabolic flux versus redox signaling may require isotopic tracing and time-resolved analyses of discrete steps in the viral life cycle.

The bidirectional redox-pharmacology data presented here highlight cellular redox homeostasis as a tractable target for host-directed PRRSV intervention. Three mechanistic entry points emerge from our work. First, broad-spectrum thiol antioxidants (NAC and exogenous GSH) and mitochondrial-targeted antioxidants (Mito-TEMPO and conceptually Mitoquinone) suppressed PRRSV replication in both PHGDH-replete and PHGDH-deficient cells, with no detectable cytotoxicity at the doses used. These compounds have established safety profiles in mammals and are attractive candidates for adjunct evaluation *in vivo*. Second, because the antiviral activity of PHGDH is independent of its serine-synthesis catalysis, therapeutic strategies should focus on preserving PHGDH protein abundance and its non-canonical mitochondrial/scaffolding functions rather than on supplying its catalytic substrates; this could include preventing the PRRSV-induced downregulation of PHGDH, stabilizing mitochondrial PHGDH, or restoring downstream antioxidant programs. Third, because PRRSV itself downregulates PHGDH (Fig. S1E through G), agents that prevent or reverse PHGDH suppression could disable an otherwise proviral metabolic program. Importantly, host-directed strategies targeting redox homeostasis are unlikely to elicit classical viral genetic resistance and could complement vaccine-mediated control of the genetically diverse PRRSV strains.

Our study has several limitations. First, while we observed altered NAD(H), NADP(H), and glutathione redox ratios, direct measurement of serine/glycine flux and one-carbon pathway activity would strengthen the mechanistic link between PHGDH enzymatic function and redox buffering. Second, the precise stages of the PRRSV life cycle enhanced by oxidative stress remain to be defined. Third, PHGDH depletion caused mitochondrial injury that may impact cell physiology beyond redox regulation; careful controls for cytotoxicity and cell cycle effects will be important in follow-up studies. Fourth, although PRRSV infection downregulates PHGDH at both the mRNA and protein levels, the viral proteins responsible for this suppression and the underlying mechanism remain to be identified, which will be addressed in future studies.

**Fig 8:**
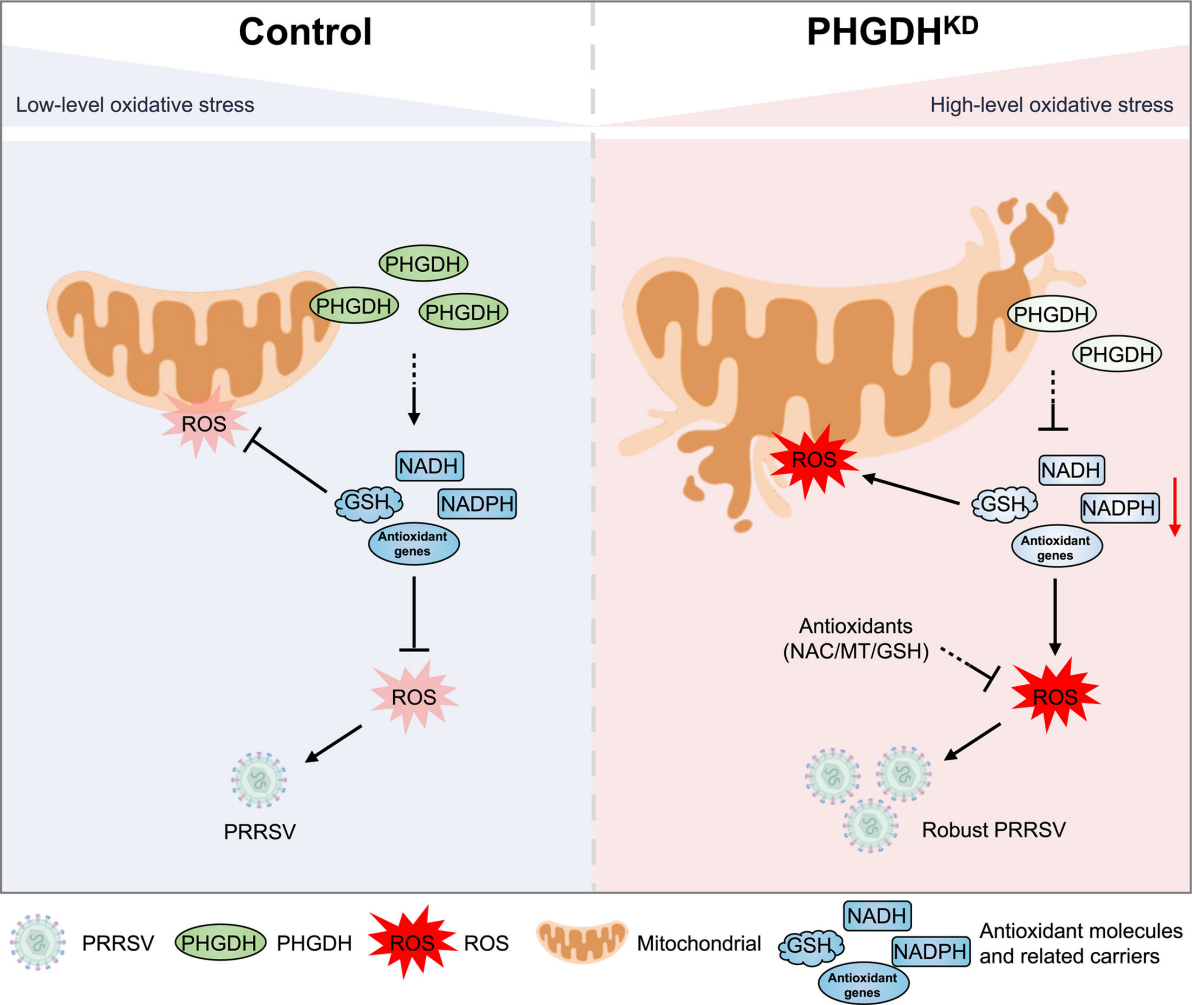
A model of the PHGDH–redox axis in PRRSV infection. Schematic summarizing the proposed mechanism: in control cells, PHGDH supports antioxidant gene programs and maintains reducing equivalents (GSH, NADH, and NADPH) to limit ROS/mROS, thereby restraining PRRSV replication. Upon PHGDH depletion, antioxidant genes and reducing equivalents decline, ROS/mROS accumulate, and PRRSV replication is enhanced.

In summary, we propose a model in which PHGDH restricts PRRSV by maintaining antioxidant gene expression and reducing equivalent availability, thereby limiting ROS/mROS accumulation that otherwise promotes virus replication (Fig. 8). Concurrently, PHGDH supports mitochondrial integrity through an antioxidant-independent mechanism that warrants further investigation. These findings expand our understanding of how host serine metabolism and redox homeostasis intersect with Arterivirus replication and highlight oxidative stress pathways as potential targets for host-directed PRRSV intervention.

## MATERIALS AND METHODS

### Cells and virus

MARC-145 cells were cultured in Dulbecco’s modified Eagle’s medium (DMEM) (Gibco, Waltham, MA, USA) with 10% fetal bovine serum (FBS) (Gibco, Rockville, MD, USA), penicillin (100 IU/mL), and streptomycin (100 μg/mL) (Invitrogen) at 37°C under a humidified 5% CO_2_ atmosphere. Primary PAMs were collected from 1-month-old SPF pigs (Large White) (Beijing Center for SPF Swine Breeding & Management) as previously described (35) and grown in RPMI 1640 medium (Gibco, Waltham, MA, USA) containing 10% FBS (Gibco, Rockville, MD, USA). The eighth passage virus of the Chinese HP-PRRSV strain JXwn06 (GenBank accession number: EF641008) was used in this study (36). The 50% tissue culture infectious dose (TCID_50_) of PRRSV determined by the Reed-Muench method has been reported before (37).

### Antibodies and reagents

Anti-PHGDH (ab57030) antibody was purchased from Abcam (Cambridge, UK); anti-DDDDK (PM020) antibody was purchased from MBL (Tokyo, Japan); anti-β-actin (20536-1-AP), anti-PHGDH (14719-1-AP), anti-Nrf2 (16396-1-AP), anti-HO-1 (10701-1-AP), anti-CAT (21260-1-AP), anti-SOD1 (10269-1-AP), and anti-SOD2 (24127-1-AP) antibodies were purchased from Proteintech Group, Inc. (Chicago, USA); mouse anti-PRRSV N monoclonal antibody was stored in our laboratory; horseradish peroxidase-conjugated goat anti-rabbit (ZB-2301) and anti-mouse (ZB-2305) IgG secondary antibodies were purchased from ZSGB-BIO (Beijing, China); H2DCFDA (D399), MitoSOX Red (M36008), and Alexa Fluor (488, 568) conjugated secondary antibodies were purchased from Thermo Fisher (Massachusetts, USA); GSH and GSSG Assay Kit (S0053), NAD+/NADH Assay Kit with WST-8 (S0175), NADP+/NADPH Assay Kit with WST-8 (S0179), and ATP Assay Kit (S0026) were purchased from Beyotime Biotechnology (Jiangsu, China); Lactic Acid assay kit (A019-2-1) and Glucose kit (glucose oxidase method) (A154-1-1) were purchased from Nanjing Jiancheng Bioengineering Institute. NAC (HY-134495), Mito-TEMPO (HY-112879), GSH (HY-D0187), and 2-DG (HY-13966) were purchased from MedChem Express (Monmouth Junction, New Jersey, USA).

### Plasmid construction and transfection

The recombinant plasmids were constructed as previously described (38). The coding regions of PHGDH from the *Chlorocebus sabaeus* (GenBank accession number: XM_007977361.2) were amplified with polymerase chain reaction by using the primer pairs shown in Table S1 and cDNAs from MARC-145 cells. The obtained PCR products were cloned into the pCMV-Flag-N vectors. Catalytically inactive PHGDH mutants, Flag-PHGDH-R236E and Flag-PHGDH-V425M, were generated by site-directed mutagenesis on the Flag-PHGDH-WT backbone using overlap extension PCR; the primers are listed in Table S2. All constructs were confirmed by Sanger sequencing. The recombinant plasmids were transfected into MARC-145 cells using Lipofectamine 3000 Reagent (Thermo Fisher, Waltham, MA, USA) according to the manufacturer’s instructions. Following 12 h of transfection, the medium was replaced with fresh DMEM (Gibco, Waltham, MA, USA) with 10% fetal bovine serum (Gibco, Rockville, MD, USA), and the cells were cultured in a humidified incubator containing 5% CO_2_ at 37°C until use.

### RT-qPCR

Total RNA was extracted from cells using the Cell Total RNA Isolation Kit (ZOMANBIO, China) according to the manufacturer’s instructions. Then, the RNA was reverse transcribed using FastKing cDNA First Strand Synthesis Kit (TIANGEN, China). RT-qPCR analysis was performed in triplicate using SYBR Green PCR Master Mix (Vazyme, China) on a CFX96 real-time PCR system (Bio-Rad, USA). Gene-specific primers were designed with the primer design software Primer Express 3.0, as shown in Table S2. The threshold cycle (Ct) value related to the RNA levels of each target gene was normalized to β-Actin, and the relative quantification of each sample was analyzed by the 2^(−ΔΔCt)^ method (39).

### Virus replication assay

To further investigate the role of PHGDH in PRRSV replication, MARC-145 cells cultured in 12-well plates were transfected with siPHGDH-1 for the required duration and then inoculated with PRRSV at an MOI of 0.1. After the specified time point, samples were subjected to freeze-thawing three times. Cells and the cell culture medium were harvested for virus titration, expressed as log10TCID_50_/mL, according to the Reed-Muench endpoint method. Additionally, mRNA levels of PRRSV nsp2 or N gene were determined by RT-qPCR. The expression of the PRRSV N protein in cells was detected by immunoblotting analysis as previously reported (40).

### RNA interference

The targeting sequences of siRNAs were synthesized by GenePharma (Jiangsu, China). All sequences are listed in Table S3. MARC-145 cells were transfected with 30 nM siRNA or scrambled siRNAs (siNC) using Lipofectamine RNAiMAX (Thermo Fisher, Waltham, MA, USA) according to the manufacturer’s instructions. After 24 h, the knockdown efficiency was determined by Western blot analysis.

### RNA sequencing

RNA sequencing was performed by LC-Bio Technologies Co., Ltd. (Hangzhou, China). The raw RNA sequencing data generated in this study have been deposited in the NCBI Sequence Read Archive (SRA) under the BioProject accession number PRJNA1464194.

### Flow cytometry

For the detection of intracellular ROS and mROS, cells were washed twice with PBS, detached from the culture vessel using 0.25% trypsin-EDTA, and resuspended and incubated with 10 μM H2DCFDA or 1 μM MitoSOX in DMEM containing 2% FBS at 37°C for 30 min. Cells were then centrifuged at 1,000 × *g* at 4°C for 5 min and resuspended in fresh medium. The fluorescence was detected using BD FACSVerse (BD Biosciences, USA) through the FITC or PE channel. At least 10,000 events for each sample were acquired. FlowJo software (v10.8.1) was used to analyze the data.

### Western blot analyses

The treated cells were rinsed three times with PBS and then lysed with cell lysis buffer (Beyotime P0013). The cell lysate was mixed with 5× SDS loading buffer (Epizyme LT101), resolved on 12.5% acrylamide SDS-PAGE gels (Epizyme PG213), and electroblotted onto PVDF membranes. The membranes were blocked with 5% nonfat dry milk in Tris-buffered saline containing 0.05% Tween 20 (TBST) for 2 h and incubated with the indicated antibodies at 4°C overnight. After three washes with TBST, the membranes were incubated with horseradish peroxidase-conjugated goat anti-mouse IgG or goat anti-rabbit IgG at room temperature for 1 h. Signals were visualized using a ChemiDoc Touch imaging system (Bio-Rad) and quantified using ImageJ software. For all immunoblots referenced in the manuscript, band intensities (target/ACTB) were quantified across at least three independent replicates and presented as bar graphs.

### Immunofluorescence and imaging

Before immunofluorescence, MARC-145 cells were inoculated in a 12-well plate containing a cover glass and transfected with the specified plasmids or siRNA, or inoculated with PRRSV. Then, the original medium was discarded after the specified time point and fixed with 4% paraformaldehyde in PBS at room temperature for 15 min. Next, the cells were infiltrated with 1% Triton X-100 in PBS for 10 min. Subsequently, the cells were blocked with 0.5% bovine serum albumin (BSA) in PBS and incubated with the corresponding primary antibody diluted in BSA for 1 h at room temperature. Then, the cells were incubated with the appropriate fluorescence-conjugated secondary antibodies for 1 h at room temperature. Finally, DNA was stained with DAPI, and the covered slides were observed under a fluorescence microscope (Nikon A1). The images were processed by the NIS-Elements Viewer software. The fluorescence intensity profile of images was quantified using ImageJ software.

### Transmission electron microscopy assay

Transmission electron microscopy assay was conducted as previously described (41). After being washed three times with pre-cooled PBS, the cells were scraped and centrifuged at 800 × *g* for 10 min. The cell pellets were fixed in 2.5% glutaraldehyde overnight at 4°C, post-fixed in 1% osmium tetroxide for 1 h at room temperature, and thoroughly rinsed with distilled H_₂_O prior to staining with 1% aqueous uranyl acetate for 1 h. Subsequently, the cells were dehydrated in a graded ethanol series and embedded in Eponate 12 resin (Ted Pella, Cat. No. 18010). Ultrathin sections (70–80 nm in thickness) were cut with an ultramicrotome. After staining with uranyl acetate and lead citrate, the sections were observed using an HT7800 transmission electron microscope (Hitachi, Japan).

### Statistical analysis

Statistical analyses were performed using GraphPad Prism 8.0 software (San Diego, CA, USA). For two-group comparisons, a two-tailed unpaired Student’s *t*-test was used. For comparisons across more than two groups, one-way ANOVA or multiple *t*-tests were used. The results were presented as the mean values ± standard deviation (SD) from at least three independent experiments, and *P* value below 0.05 was considered statistically significant (*P < 0.05; **P < 0.01; and ***P < 0.001).

## Data Availability

All data supporting the findings in the paper are available within the paper and its supplemental material. All relevant data are available from the authors. The raw RNA-seq data generated in this study have been deposited in the NCBI Sequence Read Archive (SRA) under the BioProject accession number PRJNA1464194.

## SUPPLEMENTAL MATERIAL

Supplemental figures and tables spectrum.00588-26-s0001.docx Figures S1 to S5 and Tables S1 to S3.

ASM does not own the copyrights to Supplemental Material that may be linked to, or accessed through, an article. The authors have granted ASM a non-exclusive, world-wide license to publish the Supplemental Material files. Please contact the corresponding author directly for reuse.
