# Supplementary material for: PHGDH restricts porcine reproductive and respiratory syndrome virus replication by sustaining cellular antioxidant capacity and redox homeostasis
Source: Microbiol Spectr. 2026 Jun 25;14(8):e00588-26. doi: 10.1128/spectrum.00588-26 (PMC13435642; doi:10.1128/spectrum.00588-26)
Supplement: Supplementary file 1 [file spectrum.00588-26-s0001.docx]

**Supplemental Material**

**Figure S1. PRRSV infection downregulates PHGDH expression and PHGDH depletion enhances PRRSV replication in both MARC-145 cells and PAMs.**

(A) MARC-145 cells were transfected with negative control siRNA (siNC) or two independent siRNAs targeting PHGDH (siPHGDH-1 and siPHGDH-2). The protein levels of PHGDH were analyzed by immunoblotting. (B–D) Primary PAMs were transfected with siNC or siPHGDH and infected with PRRSV at an MOI of 0.5 for 24 h. Intracellular PRRSV N mRNA levels were quantified by RT-qPCR (B). PHGDH and PRRSV-N protein levels were detected by Western blot (C). Band intensities from three independent experiments were quantified with ImageJ (D). (E–G) MARC-145 cells were mock-infected or infected with PRRSV at an MOI of 1 for 36 h. PHGDH mRNA levels were quantified by RT-qPCR (E). PHGDH and PRRSV-N protein levels were detected by Western blot (F). Band intensities from three independent experiments were quantified with ImageJ (G). Data are mean ± SD of three independent experiments, each dot represents one independent replicate (*n* ≥ 3). Statistical significance was assessed by two-tailed Student’s t-test, ns, not significant; * *p* < 0.05, ** *p* < 0.01.

**Figure S2. Quantifications of antioxidant proteins, Nrf2 localization, and the antiviral effect of catalytically inactive PHGDH mutants.**
(A–C) Densitometric quantification of CAT (A), SOD1 (B), and SOD2 (C) protein levels relative to ACTB in mock- and PRRSV-infected MARC-145 cells transfected with siNC or siPHGDH. (D–E) Densitometric quantification of Nrf2 (D) and HO-1 (E) protein levels relative to ACTB in PRRSV-infected MARC-145 cells transfected with siNC or siPHGDH. (F) Representative immunofluorescence images showing the subcellular localization of Nrf2 (red) and PHGDH (green) in MARC-145 cells transfected with siNC or siPHGDH. Nuclei were counterstained with DAPI (blue). Scale bar, 100 μm. (G) MARC-145 cells were transfected with empty vector (Vector), Flag-PHGDH-WT, or the catalytically inactive mutants Flag-PHGDH-R236E and Flag-PHGDH-V425M, and then infected with PRRSV. Viral N protein and Flag-PHGDH expression were detected by Western blot. (H) Densitometric quantification of PRRSV-N protein levels relative to ACTB from panel G. Data are presented as mean ± SD from three independent biological replicates. Statistical significance was assessed by two-tailed unpaired Student's t-test (for panels A-E), or one-way ANOVA (for panel H). ns, not significant; * *p* < 0.05, ** *p* < 0.01, *** *p* < 0.001.

**Figure S3.** **Imaging and gating controls support that PHGDH knockdown elevates intracellular ROS and mROS.**

(A) Representative fluorescence images of H2DCFDA staining (green) showing intracellular ROS in MARC-145 cells transfected with control siRNA (siNC) or PHGDH siRNA (siPHGDH) under basal conditions (MOCK) or after PMA stimulation (positive control). Scale bars, 500 μm. (B) Quantification of ROS fluorescence intensity (MFI, AU) corresponding to (A), comparing siNC and siPHGDH cells under MOCK and PMA conditions. (C) Representative flow cytometry density plots of H2DCFDA (FITC channel) showing intracellular ROS in siNC and siPHGDH cells with or without PRRSV infection. (D) Representative flow cytometry density plots of MitoSOX Red (PE channel) showing mitochondrial superoxide (mROS) in siNC and siPHGDH cells with or without PRRSV infection. “Positive” and “Unstained” samples were included to define the gate and background signal. Data are mean ± SD of three independent experiments, each dot represents one independent replicate (*n* ≥ 3). Statistical significance was assessed by multiple t-tests, ns, not significant; *** *p* < 0.001.

**Figure S4.** **NAC fails to rescue PHGDH knockdown-associated mitochondrial dysfunction, while PHGDH shows partial mitochondrial localization.**

(A) Mitochondrial membrane potential (Δψm) was assessed by JC-1 staining in PRRSV-infected siNC and siPHGDH cells treated with NAC; Δψm is expressed as aggregate/monomer fluorescence ratio. (B) Immunoblot analysis of DRP1 and phospho-DRP1 (Ser616) in siNC and siPHGDH cells treated with DMSO or NAC during PRRSV infection. (C) Densitometric quantification of p-DRP1/DRP1 ratios from (B). (D) Confocal microscopy showing partial co-localization of Flag-PHGDH (green) with the mitochondrial marker pDsRed-Mito (red); nuclei were stained with DAPI (blue). Scale bars, 10 μm. (E) Right panels show fluorescence intensity profiles of Flag-PHGDH (green) and pDsRed-Mito (red) across red-outlined regions, supporting co-localization. Data are mean ± SD of three independent experiments, each dot represents one independent replicate (*n* ≥ 3). Statistical significance was assessed by two-tailed Student’s t-test (for panel A) and the multiple t-tests (for panel C), * *p* < 0.05, ** *p* < 0.01.

**Figure S5. The ROS inducer PMA enhances PRRSV replication.**
(A) MARC-145 cells were treated with DMSO or 5 nM PMA and infected with PRRSV (MOI = 0.1). At 24 h post-infection, intracellular PRRSV-N and ACTB protein levels were assessed by Western blot. (B) Densitometric quantification of PRRSV-N protein levels relative to ACTB from panel A. Data are presented as mean ± SD from three independent experiments. Statistical significance was assessed by a two-tailed unpaired Student's t-test. **p* < 0.05.

**Table S1. PCR primers used for gene clone and plasmids contruction**

| Primer name | Sequence (5’-3’) |  |
| --- | --- | --- |
| Flag-PHGDH-Green monkey-F | ATGGCCATGGAGGCCCGAATGGCTTTTGCAAATCTGCGG | Construction of pCMV-Flag-PHGDH (Green monkey) |
| Flag-PHGDH-Green monkey-R | GTAGCCGGTACCTCGAGAGAGAAGTGGAACTGGAAGACCTCGGT |  |
| PHGDH-R236E-Green monkey-F | GTGGTGAACTGTGCCGAAGGAGGGATCGTG | Construction of pCMV-Flag-PHGDH-R236E |
| PHGDH-R236E-Green monkey-R | TTCGGCACAGTTCACCACACGCACCCCCTT |  |
| PHGDH-V425M-Green monkey-F | GAATGCCTCCTGGCCATGGCCCTGGCAG | Construction of pCMV-Flag-PHGDH-V425M |
| PHGDH-V425M-Green monkey-R | TGGCCAGGAGGCATTCTCCGAAGCCTTG |  |

Note: Underlined letters indicate the vector homologous sequences. F, forward; R, reverse.

**Table S2. Primers used for qPCR**

| Primer name | Sequence (5’-3’) | Application |
| --- | --- | --- |
| ACTB-sus-monkey-F | ACCACCATGTACCCAGGCAT | RT-qPCR detection of β-actin (Sus scrofa and Green monkey) |
| ACTB-sus-monkey-R | GGACTCGTCGTACTCCTGCT |  |
| PRRSV-NSP2-F | GTGGGTCGGCACCAGTTC | RT-qPCR detection of PRRSV Nsp2 RNA |
| PRRSV-NSP2-R | GACGCAGACAAATCCAGAGG |  |
| PRRSV-N-F | CAGTCAATCAGCTGTGCCAAA | RT-qPCR detection of PRRSV N RNA |
| PRRSV-N-R | ATCTGACAGGGCACAAGTTCCA |  |
| MFN1-F | TCCGCCTTTAACTTCTCGGG | RT-qPCR detection of MFN1 (Green monkey) |
| MFN1-R | ACAGTTTCTGCCATTATGTGTCTC |  |
| DRP1-F | ATGGCTGCTGACATTTCGGA | RT-qPCR detection of DRP1 (Green monkey) |
| DRP1-R | TGTTGGTAATGAACAGACTCCACA |  |
| Parkin-F | GCATGCACATGAAGTGTCCG | RT-qPCR detection of Parkin (Green monkey) |
| Parkin-R | GACACTGTGTATGCTCCCCC |  |
| ATF4-F | CAGCATAGCCCGTCTACCAG | RT-qPCR detection of ATF4 (Green monkey) |
| ATF4-R | TCTCCAGGAGGGTCGTAAGG |  |
| CAT-F | GCCAGCGACCAGATGAAGCA | RT-qPCR detection of CAT (Green monkey) |
| CAT-R | GTGGAGAACCGAACTGCGATGG |  |
| NQO1-F | CGGCAGAAGAGCACTGATCGTA | RT-qPCR detection of NQO1 (Green monkey) |
| NQO1-R | GGGACTCCAAACCACTGTAGGG |  |
| UCP2-F | TCCTAGCAGGCAGCACCACA | RT-qPCR detection of UCP2 (Green monkey) |
| UCP2-R | GTCGGCTTTCAGAAGGGCATCC |  |
| GSTP1-F | GGAAGGAGGAGGTGGTGACAGT | RT-qPCR detection of GSTP1 (Green monkey) |
| GSTP1-R | GGTGACGCAGGAAGGTATTGGA |  |
| APOE-F | CTGCGTTGCTGGTCACATTCCT | RT-qPCR detection of APOE (Green monkey) |
| APOE-R | TCCATCAGCGTCGTCAGTTCCT |  |
| SOD2-F | AGCCCTAATGGTGGTGGAGAAC | RT-qPCR detection of SOD2 (Green monkey) |
| SOD2-R | CCTGATTCGGACAAGCAGCAAT |  |
|  |  |  |
|  |  |  |

**Table S3. List of siRNA sequences**

| siRNA name | Target Sequence (5’-3’) | Application |
| --- | --- | --- |
| siNC | UUCUCCGAACGUGUCACGUTT | Negative control |
| siPHGDH-1 | GCAGAACCUUAGCAAAGAGGATT | Knock-down the PHGDH gene in Green monkey |
| siPHGDH-2 | GCGGAAAGUGCUCAUCAGUGATT |  |
| siPHGDH-pig-1 | GGAUGCAGUCCUUUGGGAUTT | Knock-down the PHGDH gene in pig |
| siPHGDH-pig-2 | GGGUGUGGACAAUGUGGAUTT |  |
